# Advances in the treatment of age-related hearing loss: from molecular mechanisms to targeted interventions

**DOI:** 10.3389/fnagi.2026.1924937

**Published:** 2026-09-01

**Authors:** Le Mao, Sijie Ma, Kun Zhu, Chao Feng, Xinyu Chen, Fangli Yang, Maoli Duan, Xiaowan Chen

**Affiliations:** 1 The First Clinical Medical College, Lanzhou University, Lanzhou, Gansu, China; 2 Department of Otolaryngology Head and Neck Surgery, The Second Hospital of Lanzhou University, Lanzhou, Gansu, China; 3 Department of Otolaryngology Head and Neck Surgery & Audiology and Neurotology, Karolinska University Hospital, Stockholm, Sweden; 4 ENT Division, Department of Clinical Science, Intervention and Technology, Karolinska Institute, Stockholm, Sweden; 5 Department of Otolaryngology Head and Neck Surgery, The First Hospital of Lanzhou University, Lanzhou, Gansu, China

**Keywords:** age-related hearing loss, cochlear aging, hair cell, inner ear, intervention strategies, mechanisms, mitochondria

## Abstract

Age-related hearing loss (ARHL) is the most common sensory disorder in older adults. Current hearing interventions, including hearing aids and cochlear implants, face limitations in accessibility and adherence. Therefore, efforts have been devoted to elucidating the molecular mechanisms underlying cochlear aging and identifying effective therapeutic targets for ARHL. This review summarizes several molecular mechanisms implicated in cochlear aging and ARHL, including autophagy dysregulation, oxidative stress, stereocilia degeneration, and dysregulation of mitochondrial dynamics. We further discuss recent advances in potential therapeutic targets and intervention strategies associated with these pathways, and provide theoretical support for targeted interventions for ARHL.

## 1 Introduction

Age-related hearing loss (ARHL), also known as presbycusis, is the most common sensory deficit among the elderly population, characterized by a progressive and age-dependent decline in binaural auditory function. According to the World Health Organization’s 2021 World Report on Hearing (Chadha et al., 2021), over 65% of people above 60 years of age experience some degree of hearing loss, and its prevalence increases exponentially with age. Beyond its severe impact on daily communication, ARHL is increasingly recognized as an important risk factor associated with cognitive decline, particularly dementia. A recent umbrella review reported that ARHL was associated with a combined risk ratio of 1.30 for cognitive impairment and 1.59 for dementia (Ying et al., 2023). Furthermore, each 10 dB increase in hearing loss severity was associated with a 16% higher risk of dementia (Yu et al., 2024). Importantly, as hearing loss has been identified as a potentially modifiable risk factor for dementia, effective management of hearing loss during midlife (ages 45–65 years) may contribute to an approximately 8% reduction in dementia risk (Loughrey, 2022). Given the profound impact of ARHL on cognition and quality of life, developing effective therapeutic strategies for ARHL is of great clinical importance.

Currently, hearing interventions for ARHL mainly rely on hearing aids (HAs) and cochlear implants (CIs). HAs are generally used for mild to moderate hearing loss (Kim et al., 2024). However, loudness recruitment, which markedly narrows the range between audibility and loudness discomfort level, restricts the maximum usable amplification of HA and often leads to sound discomfort. Besides, HAs typically provide less effective amplification for high-frequency sounds compared to low-frequency sounds. This limitation is particularly relevant to ARHL, which tends to affect high-frequency hearing first, thereby reducing the effectiveness of HAs in the early stage of ARHL. CIs can help overcome the limitations of HAs with severe to profound hearing loss (>70 dB HL) (Rasmussen et al., 2022). However, practical inconvenience in daily life and during certain medical examinations, such as MRI, together with the high cost of these devices, may hinder their uptake among older adults, especially in low- and middle-income countries.

Given the limitations of HAs and CIs, research efforts have increasingly focused on the etiologies and pathogenesis of ARHL. In recent years, substantial progress has been made in elucidating several mechanisms underlying ARHL, including autophagy, oxidative stress, stereocilia degeneration and mitochondrial dynamics. In this review, we will summarize advances in the molecular mechanisms and targeted interventions for ARHL, which may provide a theoretical basis for elucidating cochlear aging and developing promising targeted therapeutic strategies.

## 2 Autophagy dysregulation

Autophagy could clear misfolded or aggregated proteins and damaged organelles. It diminishes with age, which could lead to the accumulation of damaged proteins and mitochondria within the cochlea, thereby contributing to the onset of ARHL (Oh et al., 2020; Zou et al., 2024). Therefore, understanding the regulation of autophagy in the pathogenesis of ARHL is crucial.

**FIGURE 1:**
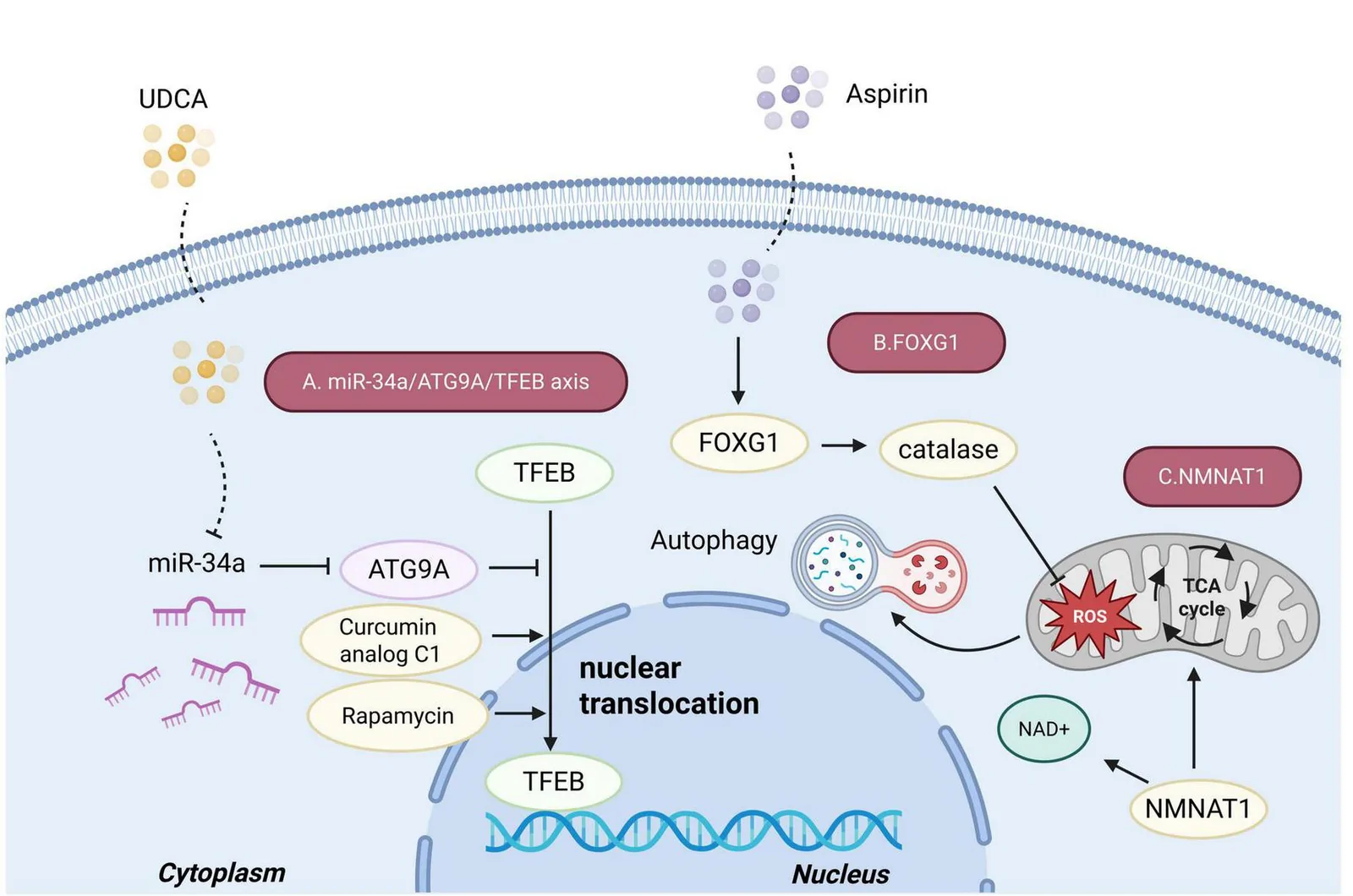
Schematic illustration of impaired autophagy in ARHL. **(A)** miR-34a (microRNA-34a) suppresses ATG9A (autophagy-related protein 9A) expression and TFEB (transcription factor EB) nuclear translocation, leading to impaired autophagy and cell death. Ursodeoxycholic acid (UDCA) reduces miR-34a expression and partially restores autophagy. Curcumin analog C1 and rapamycin increase TFEB levels in the nucleus of outer hair cells (OHCs) and autophagic flux. **(B)** FOXG1 (forkhead box G1) promotes autophagy and enhances the stress resistance of HCs. Aspirin upregulates FOXG1 and increases catalase expression while reducing reactive oxygen species (ROS) levels, thereby alleviating oxidative injury in hair cells (HCs). **(C)** NMNAT1 (nicotinamide nucleotide adenylyltransferase 1) suppresses the expression of aging-associated genes in HCs through the promotion of nicotinamide adenine dinucleotide (NAD+) synthesis, enhancement of autophagic activity, and maintenance of tricarboxylic acid (TCA) cycle metabolism. Created with BioRender.com.

Increasing evidence has demonstrated that several regulatory molecules, such as microRNA-34a (miR-34a), forkhead box G1 (FOXG1), and nicotinamide nucleotide adenylyltransferase 1 (NMNAT1), are involved in cochlear hair cell (HC) aging by impairing autophagy (Figure 1).

Most studies support the view that impaired autophagy contributes to ARHL, whereas recent evidence also suggests that excessive autophagy activation may likewise be detrimental (Ding et al., 2025). In senescent HEI-OC1 (House Ear Institute-Organ of Corti 1) cells and 12-month-old C57BL/6J mice, dual leucine zipper kinase (DLK), phosphorylated c-Jun N-terminal kinase (p-JNK) and JNK3 expression increased, which was accompanied by excessive autophagy activation. Inhibition of the DLK/JNK3 pathway reduced autophagy activation and senescence in HEI-OC1 cells, and alleviated cochlear damage and hearing loss in C57BL/6J mice, suggesting that excessive autophagy activation may accelerate ARHL progression in specific settings.

### 2.1 miR-34a/ATG9A/TFEB axis

Previous studies have shown that many microRNAs are stably expressed in the inner ear and contribute to the development, homeostasis, and aging-related pathological processes of cochlear sensory epithelium (Jiang et al., 2022; Koffler-Brill et al., 2023; Ma et al., 2023). Among them, miR-34a is closely associated with autophagy, senescence, cell cycle arrest and cell death (Liu et al., 2012; Yang et al., 2013).

Pang et al. (2017) reported that in the cochleae of 12-month-old C57BL/6 mice, miR-34a expression was significantly upregulated (*P* < 0.001, one-way ANOVA) accompanied by impairment of autophagic flux, as evidenced by decreased expression of microtubule-associated proteins 1A/1B light chain 3 (MAP1LC3B/LC3B) and accumulation of SQSTM1/p62. LC3B is widely used as a marker of autophagosome formation, while p62 commonly serves as an index of autophagic degradation (Klionsky et al., 2021). Bidirectional dysregulation of LC3B and p62, whether increased or decreased, may disrupt autophagic homeostasis in cochlear HCs, thereby impairing hair cell survival and auditory function. In HEI-OC1 cells, miR-34a overexpression resulted in a significant decrease in autophagy-related protein 9A (ATG9A) expression (*P* < 0.001, Student’s *t*-test) and impaired autophagosome-lysosome fusion, leading to cell death subsequently. Knockdown of ATG9A in HEI-OC1 cells inhibited autophagy flux and led to the blockage of autophagosome-lysosome fusion, which was similar to the effects of miR-34a overexpression, suggesting that autophagy may be regulated by miR-34a through ATG9A. Besides, a miR-34a inhibitor, ursodeoxycholic acid (UDCA) significantly reduced miR-34a-induced cell death in HEI-OC1 cells by restoring ATG9A expression and autophagic activity (*P* < 0.001, one-way ANOVA). Taurine-conjugated derivative of UDCA, tauroursodeoxycholic acid (TUDCA), has also shown protective effects in several auditory models, including *Cdh23^*erl*/*erl*^* mice (a mouse model of human autosomal recessive nonsyndromic deafness), gentamicin-treated HEI-OC1 cells and cochlear explants, and a rat model of cisplatin-induced hearing loss (Hu et al., 2016; Jia et al., 2018; Lee et al., 2020). Nevertheless, whether UDCA itself exerts protective effects for hearing loss *in vivo* remains to be determined.

They also revealed that nuclear translocation of transcription factor EB (TFEB), a master transcription factor for lysosomal biogenesis and autophagy (Settembre et al., 2011), was regulated by miR-34a in HEI-OC1 cells (Xiong et al., 2022). Treatment with rapamycin rescued the inhibition of TFEB nuclear translocation, restored cellular autophagic flux, and decreased cell death in HEI-OC1 cells. Furthermore, after 8 months of dietary rapamycin supplementation starting at 2 months of age, 10-month-old C57BL/6 mice exhibited a partial restoration of nuclear TFEB localization and autophagic flux in outer hair cells (OHCs). Loss of OHCs and inner hair cell (IHC) synaptic ribbons was attenuated, and auditory brainstem response (ABR) threshold shifts at 4, 8, and 32 kHz were significantly reduced (4 kHz: *P =* 0.025; 8 kHz: *P =* 0.007; 32 kHz: *P =* 0.025, Student’s *t*-test). Similar results (He et al., 2023) were obtained in 12-month-old C57BL/6 mice treated for 6 months at 6 months of age with a TFEB activator, curcumin analog C1. The nuclear localization of TFEB and autophagic flux increased in the OHCs, while loss of IHCs and OHCs was decreased. Moreover, ABR threshold shifts measured at 8, 16, and 32 kHz were average reduced by 22, 38, and 21 dB, respectively.

Taken together, these results indicated that miR-34a may impair autophagy by inhibiting TFEB nuclear translocation via ATG9A, thereby leading to the development of ARHL. Supplementation with UDCA to inhibit miR-34a, as well as oral administration of rapamycin for 8 months or curcumin analog C1 for 6 months to promote TFEB nuclear localization in OHCs, may represent potential therapeutic strategies for ARHL.

### 2.2 FOXG1

FOXG1 is an important nuclear transcription factor, which is widely expressed in HCs, supporting cells (SCs), marginal cells, and Hensen’s cells in the organ of Corti (Ding et al., 2020). It plays essential roles in inner ear development and hair cell survival, and is also involved in mitochondrial ATP synthesis and metabolic regulation (Jerome et al., 2022). Recently, increasing attention has been paid to the role of FOXG1 in autophagy. In Alzheimer’s disease, FOXG1 overexpression could activate autophagy through the AMPK/mTOR signaling pathway (Yun et al., 2024).

In a study by He et al., (2021), FOXG1 expression in D-galactose (D-gal)-induced senescent HEI-OC1 cells showed dynamic changes that were consistent with autophagic flux. FOXG1 and LC3B expression was markedly increased in cells treated with lower concentrations of D-gal, but both decreased significantly after treatment with higher concentrations (*P* < 0.05, one-way ANOVA). Therefore, FOXG1 may participate in a compensatory protective response during the early stage of senescence, but becomes impaired during the later decompensatory stage. In HEI-OC1 cells, downregulation of FOXG1 decreased the number of autophagosomes, autolysosomes and autophagic activity. This was accompanied by increased reactive oxygen species (ROS) and apoptosis.

Aspirin has been shown to induce autophagy in breast cancer by activating AMPK and inhibiting mTORC1 signaling (Henry et al., 2017). In D-gal-induced senescent HEI-OC1 cells treated with low doses of aspirin, FOXG1 was upregulated and autophagy levels were increased. In D-gal-induced senescent LC3-GFP mice, intraperitoneal administration of aspirin for 14 days beginning at postnatal day 70 (P70) upregulated FOXG1 and autophagy levels, while reducing the loss of IHCs and OHCs. These findings demonstrated that aspirin may reduce the loss of HCs by increasing FOXG1 expression and autophagy in cochlear HCs. Moreover, the daily injection with aspirin for 42 days starting at P42 in C57BL/6 mice exhibited increased catalase levels and decreased ROS levels in IHCs and OHCs at P90, indicating that aspirin could also reduce oxidative damage in the cochlea.

These findings supported the protective role of FOXG1 by regulating autophagy in aging HCs.Aspirin may delay HC aging by upregulating FOXG1 expression and autophagy levels in the cochlea, which is consistent with its previously reported protective effects against cochlear injury (Mazurek et al., 2012; Sha et al., 2006). However, there was no assessment of changes in hearing. Thus, the evidence is more convincing for a hair-cell protective effect than hearing improvement. Further efforts should be directed toward evaluating the effects of FOXG1 and aspirin on hearing rescue in animal models, which may facilitate the clinical translation of this potential therapeutic target.

### 2.3 NMNAT1

NMNAT1, a key enzyme in the biosynthesis of nicotinamide adenine dinucleotide (NAD+), is involved in maintaining intracellular NAD+ levels and the NAD+/NADH ratio, whereas augmenting NAD+ ameliorates cochlear damage and auditory dysfunction (Okur et al., 2023). Moreover, hNMNAT1 overexpression was able to improve autophagic flux and eliminate amyloid plaques in a drosophila model of Alzheimer’s disease (Zhu et al., 2022).

Wei et al., (2026) observed that D-gal-induced senescent HEI-OC1 cells and cochlear explants (basilar membranes from FVB mice at P3) exhibited reduced NAD+ levels and NMNAT1 expression, and impaired autophagy. NMNAT1 was significantly down-regulated in the OHCs of the apical, middle, and basal turns of cochlear explants (*P* < 0.001, one-sample *t*-test). These indicated that the downregulation of NMNAT1 might be associated with autophagy impairment. NMNAT1 overexpression in HEI-OC1 cells and cochlear explants could ameliorate D-gal-induced degeneration of the HCs by restoring autophagy flux. Notably, metabolomic analysis showed that the tricarboxylic acid (TCA) cycle and the pentose phosphate pathway were disrupted in NMNAT1-knockout HEI-OC1 cells, indicating that NMNAT1 is involved in regulating autophagy and metabolic pathways of HCs.

Available evidence suggests that NMNAT1 may be associated with HC aging and ARHL through NAD+ maintenance, protective autophagy enhancement, and TCA cycle regulation. However, current studies are largely restricted to HEI-OC1 cells and cochlear explants, which fail to fully capture the physiological complexity of ARHL. Consequently, future research should leverage *in vivo* animal models to comprehensively elucidate the precise role of NMNAT1 in ARHL.

Overall, we are more inclined to conclude that impaired autophagy, rather than excessive autophagy activation, plays a dominant role in the progression of ARHL. This view is mainly supported by the fact that most studies consistently demonstrate reduced autophagic activity and impaired autophagic flux in aging cochlear cells, whereas restoration of autophagy generally exerts protective effects on HCs aging and hearing rescue. In particular, studies targeting the miR-34a/ATG9A/TFEB axis have provided relatively strong evidence, as pharmacological interventions such as UDCA, rapamycin, and curcumin analog C1 were able to restore autophagic flux, attenuate HCs loss, and partially prevent hearing loss in mouse models. In contrast, excessive autophagy activation may represent a maladaptive response under specific stress conditions rather than the dominant mechanism.

As mentioned above, inhibition of the miR-34a/ATG9A/TFEB axis has emerged as a promising therapeutic strategy for ARHL. By comparison, although FOXG1 and NMNAT1 also appear to be involved in the regulation of protective autophagy in cochlear HCs, current evidence is less complete than the miR-34a/ATG9A/TFEB axis. The therapeutic potential of FOXG1 and NMNAT1-related pathways still requires further validation, particularly with respect to hearing preservation in animal models.

## 3 Oxidative stress

Oxidative stress is cellular oxidative damage caused by excessive ROS production or insufficient ROS clearance. The gradual accumulation of ROS in the cochlea with the simultaneous decrease in antioxidant capacity during aging causes DNA damage, mitochondrial dysfunction, apoptosis, and HCs degeneration, ultimately promoting the progression of ARHL (Xu and Yang, 2025).

**FIGURE 2:**
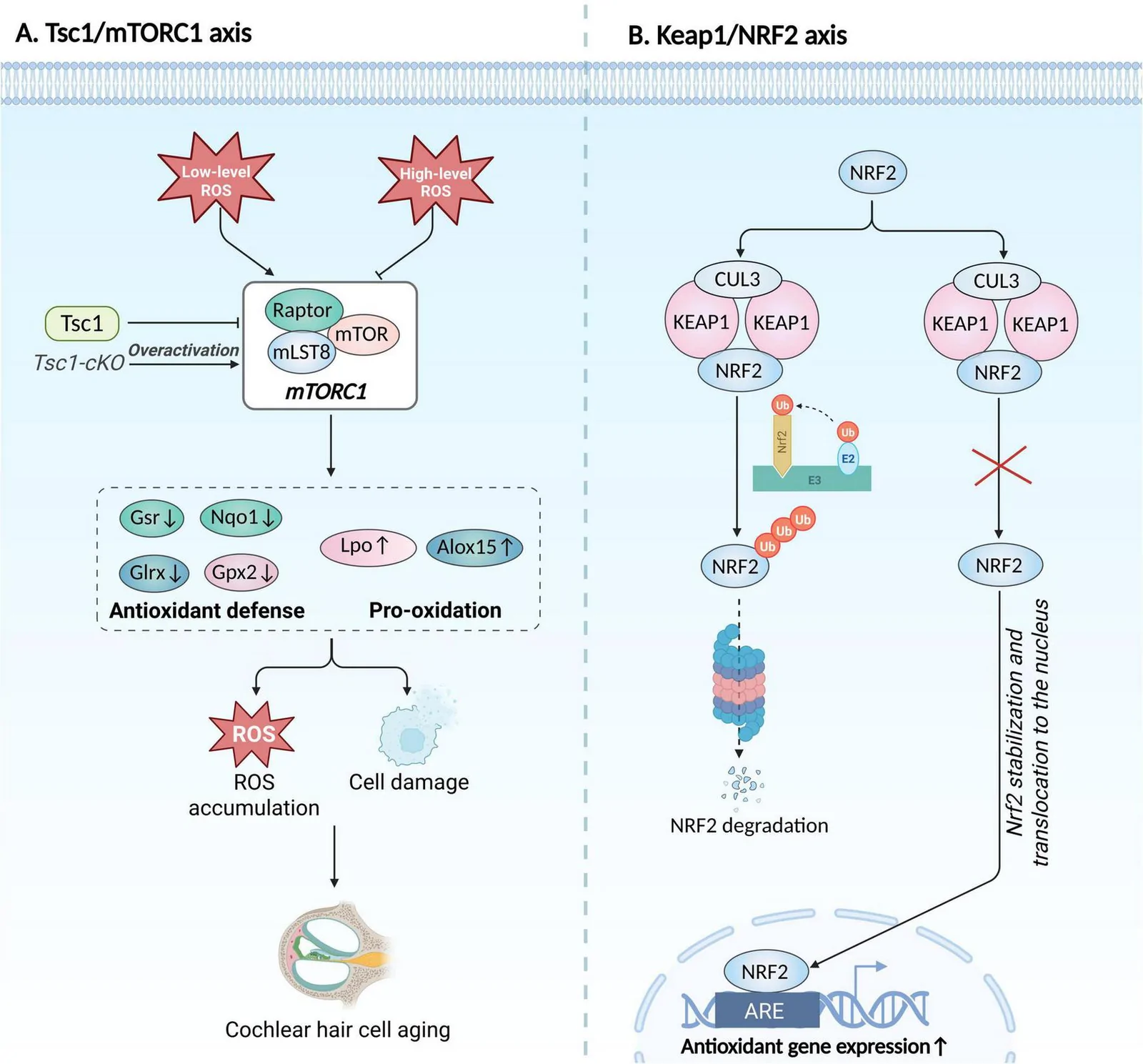
Schematic illustration of oxidative stress in ARHL. **(A)** Low-level ROS may activate mTORC1 (mammalian target of rapamycin complex 1), whereas high-level ROS may inhibit mTORC1. TSC1 (tuberous sclerosis complex 1) deficiency causes mTORC1 overactivation, impairs antioxidant defense, upregulates pro-oxidant genes, and promotes ROS accumulation and cochlear hair cell damage. **(B)** Under basal conditions, NRF2 (nuclear factor erythroid 2-related factor 2) is ubiquitinated and degraded. Inhibition of KEAP1 (Kelch-like ECH-associated protein 1) stabilizes NRF2 and facilitates its nuclear translocation, resulting in enhancing antioxidant defense and protecting cells against oxidative injury in the cochlea. Created with BioRender.com.

The abnormal activation of tuberous sclerosis complex 1/mammalian target of rapamycin complex 1 (TSC1/mTORC1) axis, as well as an impaired antioxidant response mediated by Kelch-like ECH-associated protein 1/nuclear factor erythroid 2-related factor 2 (KEAP1/NRF2) axis, may play a role in the oxidative damage underlying ARHL. The former exacerbates oxidative injury, while the latter inhibits the clearance of reactive species resulting from oxidative stress (Figure 2).

### 3.1 TSC1/mTORC1 axis

mTORC1 is a multiprotein complex consisting of mTOR, Raptor, and mLST8, which plays a role in cell growth, cellular metabolism, and stress responses (Zhang et al., 2025). Recently, a bidirectional link between oxidative stress and mTORC1 has been identified. On the one hand, ROS could stimulate mTORC1 in low doses but inhibit it in higher doses or with longer exposure (Li et al., 2010). On the other hand, excessive activation of mTORC1 could inhibit oxidative stress defense, increase the accumulation of ROS, and hence lead to cell injury (Glorieux et al., 2025). mTORC1 is negatively regulated by TSC1, and loss of TSC1 increases mTORC1 activity (Fitzian et al., 2021).

Fu et al., (2018) observed that mTORC1 was highly and specifically activated in the cochlear neurosensory epithelium (NSE) of 12-month-old C57BL/6J mice, particularly in OHCs and Deiters’ cells. They generated NSE *Raptor* conditional knockout mice (*Raptor*-cKO mice). From 3 months to 17 months of age, this model showed that the mean ABR threshold increased only slightly (∼15 dB). In agreement with the hearing test results, at 17 months of age, only minor loss of OHCs was observed. In contrast, age-matched C57BL/6 mice exhibited a dramatic increase in mean ABR threshold (>40 dB) and a significant loss of HCs, particularly OHCs (*P* < 0.001, one-way ANOVA).

They also produced NSE *Tsc1* conditional knockout mice (*Tsc1*-cKO mice) which exhibited overactivation of mTORC1 signaling. By measuring the ABR thresholds, early-onset progressive hearing loss in *Tsc1*-cKO mice was observed. At 2 weeks of age, the ABR thresholds were 20 ± 5 dB, but increased rapidly and reached 65 ± 5 dB by the age of 8 weeks. Besides, at 4 months, most OHCs in the entire cochlear epithelium were lost. At 8 months of age, IHC loss was severe, with the few remaining IHCs showing marked stereocilia fusion. In contrast, age-matched C57BL/6 mice exhibited no evident structural abnormalities in HCs.

In addition, at P30, the ROS markers 4-hydroxynonenal and 3-nitrotyrosine were dramatically elevated in OHCs, IHCs, and SCs of *Tsc1*-cKO mice. The expression of several antioxidant enzyme-related genes (*Gpx2*, *Gsr*, Nqo1, and *Glrx*) was downregulated, and the expression of several pro-oxidant genes (*Lpo* and *Alox15*) was upregulated in IHCs. These findings suggested that mTORC1 overactivation caused cochlear damage by disrupting the redox balance in the cochlea. Notably, mitochondrial defects in OHCs were not detected until P50. In contrast, OHCs displayed structural abnormalities of peroxisomes, such as multiple crystal nuclei and diffuse membranes, as early as P30. This suggested that peroxisomes, not mitochondria, may be the initial organelles in cochlear hair cells targeted by the TSC1/mTORC1 axis to modulate redox homeostasis.

Either early treatment of *Tsc1*-cKO mice with the mTORC1 inhibitor rapamycin (from P14 for 5 months) or the antioxidant N-acetylcysteine (NAC) (from P14 for 2 months) could delay the progression of hearing loss and reduce the loss of OHCs.

Considering that C57BL/6 mice may carry the recessive early-onset hearing loss susceptibility allele (*Cdh23*^753A^) (Johnson et al., 2017), they sequenced this gene in Raptor-cKO mice and Tsc1-cKO mice. However, they did not completely rule out the potential contribution of this allele to the age-related auditory phenotype. These results suggested that overactive mTORC1 signaling may participate in the progression of ARHL by disrupting the redox balance in cochlear HCs. Treatment with rapamycin or NAC may be potential therapeutic strategies for ARHL. Additionally, given the early onset of peroxisomal alterations, future studies should distinguish the role of peroxisomes from that of mitochondria in ARHL.

### 3.2 KEAP1/NRF2 axis

NRF2 is a central transcription factor of the cellular antioxidant defense system. Under physiological conditions, NRF2 is ubiquitinated in the cytoplasm by the KEAP1-CUL3 E3 ubiquitin ligase complex, leading to its degradation. Upon exposure to ROS, which inactivate KEAP1, NRF2 becomes stabilized and subsequently translocates into the nucleus, where it binds to antioxidant response elements (ARE) and activates multiple antioxidant and cytoprotective genes (Baird and Yamamoto, 2020).

Oishi et al., (2020) observed increased expression of multiple NRF2 target genes in the whole cochlea of 12-month-old *Keap1*-knockdown (*Keap1^*FA*/*FA*^*) mice compared with age-matched C57BL/6 mice. Furthermore, levels of oxidative stress markers 4-hydroxy-2-nonenal and 8-hydroxydeoxyguanosine were reduced, degeneration of spiral ganglion neurons (SGNs) and loss of OHCs at the apical and middle turns of the cochlea were markedly ameliorated (*P* < 0.01, Student’s *t*-test). ABR thresholds in the 12-month-old *Keap1^*FA*/*FA*^* mice, especially at low-mid frequencies (4, 8, and 12 kHz), were significantly lower (∼20 dB, *P* < 0.05, Student’s *t*-test) than age-matched C57BL/6 mice. Notably, there were no significant differences in the activity of NRF2 pathway among 2-, 5- and 12-month-old C57BL/6 mice, suggesting that during physiological aging, the endogenous NRF2 pathway in the cochlea was not fully activated in response to the accumulation of ROS. The antioxidant system alone may be insufficient to counteract age-related oxidative damage. Conversely, NRF2 pathway activation by KEAP1 inhibition could ameliorate the loss of OHCs and the degeneration of SGNs by strengthening the antioxidant system and attenuating the oxidative damage of ROS, thereby delaying the progression of cochlea aging and ARHL.

In addition, they genotyped their C57BL/6-background mice and confirmed that both *Keap1^*FA*/*FA*^* mice and wild-type mice were homozygous for the *Cdh23* allele. This design reduced the risk that unequal *Cdh23* genotypes caused differences between groups and therefore supports a protective effect of NRF2 activation in C57BL/6-background mice.

However, this study primarily demonstrated the protective effect of activating NRF2 pathway by knocking down *Keap1*, without directly tracking the dynamics of NRF2 nuclear translocation within cochlear cells. In addition, since the phenotype caused by Keap1 knockdown could generally be reversed by simultaneous NRF2 inhibition, it was still necessary to disrupt *Nrf2* gene in *Keap1^*FA*/*FA*^* mice to further verify the direct role of *Nrf2* gene in ARHL.

From our perspective, the TSC1/mTORC1 axis and the KEAP1/NRF2 axis do not appear to represent conflicting mechanisms, but rather two interconnected processes, namely enhanced oxidative injury and insufficient antioxidant defense. The role of TSC1/mTORC1 overactivation is supported more directly by animal studies showing pronounced hearing phenotypes and partial rescue by rapamycin or NAC. The findings on KEAP1/NRF2 demonstrate that the endogenous antioxidant response in the aging cochlea is insufficient to counteract accumulated ROS. In conclusion, oxidative stress in ARHL is more likely to arise from the combined effects of increased oxidative burden and inadequate antioxidant defense.

## 4 Stereocilia degeneration

**FIGURE 3:**
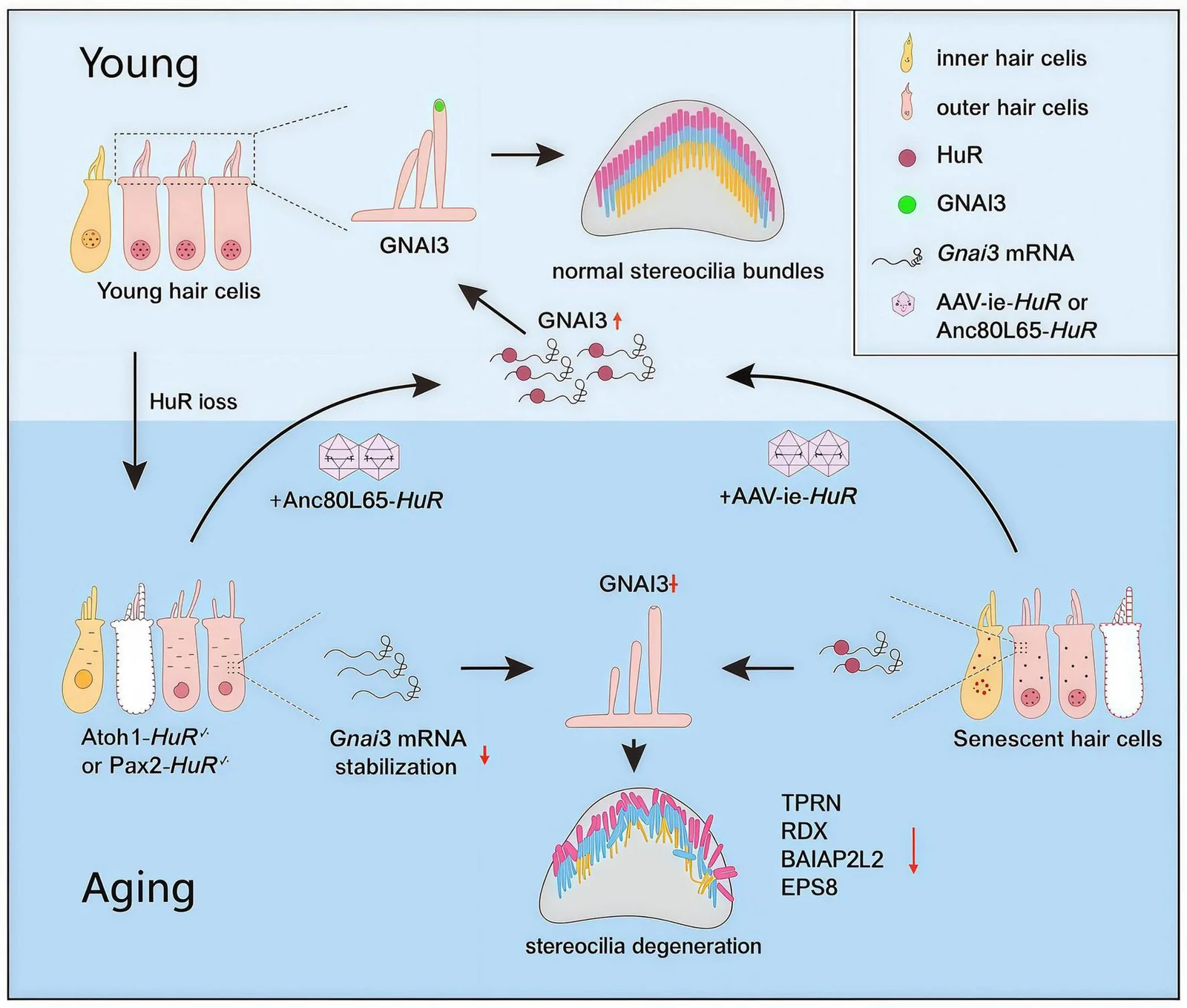
Schematic illustration of stereocilia degeneration in ARHL. Reproduced from Ref. (Guo et al., 2025) with permission from Springer Nature. Young hair cells have HuR (Human antigen R), *Gnai3* (G protein subunit alpha i3), normal stereocilia bundles. In HuR-deficient mice (Atoh1-HuR^–/–^ and Pax2-HuR^–/–^), HuR loss leads to reduced *Gnai3* mRNA stabilization, reduced *Gnai3*, and causes stereocilia degeneration in OHCs. AAV-ie-HuR or Anc80L65-HuR by HuR overexpression in HCs restores *Gnai3* expression and delays hearing loss.

The stereocilia in HCs are highly organized and necessary for the cochlear mechanoelectrical transduction (MET). Defects in the stereocilia, including disorganization, fusion, and loss, can damage MET and HCs, contributing to hearing loss (Vélez-Ortega et al., 2017). In aging mouse models, changes in the stereocilia have been observed, but the underlying mechanisms remain unclear (Figure 3).

Human antigen R (HuR), a classical RNA-binding protein, is involved in the stability, transcription, and translation of target mRNAs (Govindaraju and Lee, 2013). Guo et al., (2025) found that HuR expression increased with age in the mouse cochlea in C57BL/6J mice by searching a single-cell RNA sequencing atlas. Notably, in 1-month-old mice, the HuR protein was exclusively expressed in the nucleus of HCs. But amount of cytoplasmic HuR localization was also observed in the OHCs of 12-month-old mice and macaca fascicularis aged 16–18 years. This demonstrated that C57BL/6J mice and macaca fascicularis exhibited the obvious translocation of HuR from the nucleus to the cytoplasm in the HCs, especially OHCs.

They generated two types of mutant mice, namely Atoh1-HuR^–/–^ mice, in which the HuR gene was deleted in HCs alone, and Pax2-HuR^–/–^ mice, in which the HuR gene was deleted in the entire cochlea. At P30, hearing was mostly normal in both types of mice. But at P45 and P60, the mice showed significantly high frequencies (16, 24 and 32 kHz) hearing loss (*P* < 0.001, one-way ANOVA) with almost complete deafness observed at P90. Besides, at P60, both groups of mutant mice exhibited a significant loss of HCs, especially OHCs and stereocilia degeneration, including disorganization, internalization, and fusion. The MET function defect in these HuR-deficient mice was observed by FM1-43 uptake assay in HCs. Furthermore, at P3, anc80L65-mediated HuR overexpression decreased loss of OHCs and partially restored hearing at P90, most effectively at the middle and high frequencies.

In addition, they also generated the conditional ablation of *HuR* in SCs (Sox2-*HuR^–/–^*) by crossing *HuRf^*lox*/*flox*^* mice with Sox2-Cre*^ER^* mice and administering tamoxifen for 5 days at P21. Hearing function remained normal in this model at P90. This further confirmed HCs may be the only cell type affected by *HuR* deficiency.

Using RNA immunoprecipitation sequencing, they identified G protein subunit alpha i3 (*Gnai3*), a HuR target which has been shown that be associated with the function of stereocilia and the survival of HCs (Beer-Hammer et al., 2018). At P3, *Gnai3* was overexpression via Anc80L65- *Gnai3* injection in Atoh1-*HuR^–/–^* mice and Pax2-*HuR^–/–^* mice. At P90, hearing loss and loss of OHCs were partially rescued, supporting the idea that *Gnai3* overexpression may rescue auditory function in *HuR*-deficient mice. Expression of several genes, including those encoding stereocilia-associated proteins (*Pjvk*, *Grxcr2*, *Myo3a* and *Myo15*) and those involved in MET (*Loxhd1*, *Tomt* and *Tmc1*),was downregulated in Pax2-HuR^–/–^ mice.

Considering all of these findings, HuR may delay the progression of ARHL by maintaining the stability of *Gnai3* mRNA and the structure of stereocilia in HCs. The available data are relatively convincing, as both loss-of-function and rescue experiments consistently link *HuR*-deficiency to stereocilia degeneration, MET dysfunction, HC loss, and hearing impairment. Therefore, HuR may represent a potential therapeutic target for ARHL. However, several important issues remain unresolved. First, the relative contribution of increased HuR expression and age-related HuR translocation from the nucleus to the cytoplasm remains unclear. Second, most evidence was obtained from the rapidly aging SAMP8 mice rather than from physiological aging mice, which may not fully recapitulate the natural course of late-stage ARHL. Accordingly, expanding this study to natural aging mice may enhance the generalizability of the results.

## 5 Dysregulation of mitochondrial dynamics

HCs are highly energy-demanding cells and depend on mitochondria-generated energy for their normal cellular physiology. As a result, they are especially sensitive to mitochondrial damage (O’Sullivan et al., 2023).

**FIGURE 4:**
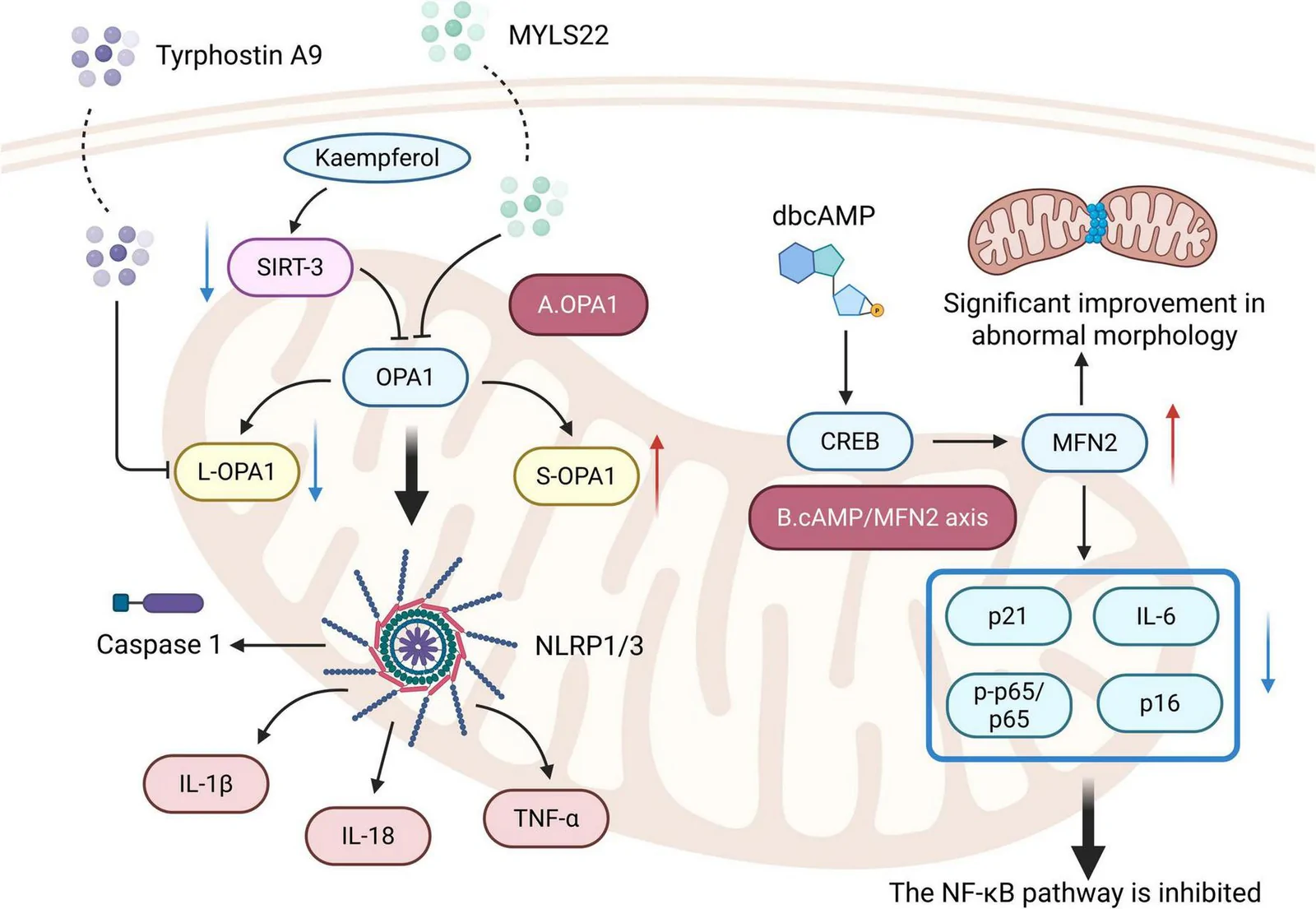
Schematic illustration of dysregulation of mitochondrial dynamics in ARHL. **(A)** OPA1 (optic atrophy protein 1) dysregulation, including excessive L-OPA1 (long-form OPA1) cleavage and increased acetylation, impairs mitochondrial fusion, promotes mitochondrial fragmentation, and trigger NLRP1/3-caspase-1 inflammatory pathway, ultimately accelerating HC aging. SIRT3 (Sirtuin 3)-mediated OPA1 activation exerts a protective effect against cochlear aging. **(B)** Activation of the cAMP/MFN2 (mitofusin 2) axis enhances MFN2-dependent mitochondrial fusion, improves mitochondrial morphology, and inhibits HC aging and NF-κB inflammatory pathway. Created with BioRender.com.

Mitochondria continuously undergo fusion and fission to adapt to metabolic demands and cellular stress, a process collectively referred to as mitochondrial dynamics (Tábara et al., 2025). Mitofusin 1/2 (MFN1/2) is required on adjacent mitochondria to mediate outer membrane fusion, and inner membrane fusion is mainly mediated by optic atrophy protein 1 (OPA1) (Figure 4).

### 5.1 OPA1

OPA1 is localized in the inner mitochondrial membrane and plays a key role in maintaining mitochondrial cristae integrity. Its activity is mainly regulated by the proportion of long-form OPA1 (L-OPA1) and deacetylation status (Nyenhuis et al., 2023). Under mitochondrial stress, OPA1 could be excessively cleaved by proteases into short-form OPA1 (S-OPA1). A reduced L-/S-OPA1 ratio impairs mitochondrial fusion and induces fragmentation (Muñoz et al., 2023). Sirtuin 3 (SIRT3) is a mitochondrial deacetylase, and its downregulation results in OPA1 hyperacetylation and reduced OPA1 activity, thereby exacerbating mitochondrial dynamics dysregulation (Zhang et al., 2020).

Zhang et al., (2024) reported that, in D-gal-induced senescent HEI-OC1 cells, a gradual transition of the mitochondria from normal elongated forms to fragmented punctate structures, which was accompanied by excessive cleavage of L-OPA1 and increased OPA1 acetylation, suggesting that the dysregulation of mitochondrial dynamics may be associated with abnormalities in both the abundance and activity of OPA1. Treating senescent HEI-OC1 cells with either a specific OPA1 inhibitor (MYLS22) or a specific L-OPA1 inhibitor (Tyrphostin A9) further aggravated mitochondrial fragmentation and increased the proportion of senescent cells. These findings indicated that inhibition of L-OPA1 may disrupt mitochondrial dynamics and accelerate HC aging. In contrast, SIRT3 activation with kaempferol, a flavonoid antioxidant, significantly increased OPA1 activity. A significant improvement of mitochondrial morphology and a decreased proportion of senescent cells were observed (*P* < 0.001, one-way ANOVA). These indicated a protective role of SIRT3-mediated OPA1 activation in HC aging.

In addition, they generated a mouse model with increased SIRT3 activity (SIRT3 K223R mice) by CRISPR/Cas9 mediated gene editing. At 24 months of age, K223R mice had normal mitochondrial morphology, orderly and distinct mitochondrial cristae structure, as well as more preserved OHCs, IHCs, and SGNs than age-matched C57BL/6 mice. In contrast, age-matched C57BL/6 mice showed obvious mitochondrial abnormalities, including mitochondrial swelling, cristae disruption or loss, mitochondrial fragmentation, and vacuolization, together with loss of OHCs and SGNs throughout the entire cochlea and loss of IHCs in the basal turn. Besides, ABR thresholds in 6-month-old and 24-month-old K223R mice were significantly lower than those in age-matched C57BL/6 mice. These findings suggested that activation of SIRT3 could delay cochlear degeneration and ARHL.

They also observed that the NLRP1 and NLRP3 inflammasomes were activated in senescent HEI-OC1 cells, along with the up-regulated expression of caspase-1, IL-1β, IL-18, and TNF-α.

These findings supported that quantities and the acetylation activity of OPA1 may play important roles in the dysregulation of mitochondrial dynamics, which may further activate NLRP1/3-caspase-1 inflammatory pathway and accelerate HC aging and ARHL. Notably, although OPA1 dysfunction was associated with activation of the NLRP1/3-caspase-1inflammatorypathway, the precise mechanisms have not yet been fully elucidated.

### 5.2 cAMP/MFN2 axis

MFN2 is a GTPase localized in the outer mitochondrial membrane, where it plays a supportive role in mediating mitochondrial fusion (Zanfardino et al., 2025). Activation of cAMP-response element-binding protein (CREB), a major downstream target of cAMP, could upregulate MFN2 expression (Li et al., 2020).

Liu et al., (2025) found that, after addition with a cAMP analog dibutyryl-cAMP (dbcAMP) in D-gal-induced senescent HEI-OC1 cells, MFN2 expression was significantly increased. Abnormal mitochondrial morphology, including fragmentation, shortened branch length, and a reduced aspect ratio, was markedly improved, and the formation of elongated and interconnected mitochondrial networks was promoted. At the same time, the expression of senescence-associated markers, including *Cdkn1a* (*p21*), *Cdkn2a* (*p16*), IL-6, and TNF, was reduced, and the p-p65/p65 ratio was decreased, suggesting that the NF-κB inflammatory pathway was suppressed. After siRNA-mediated knockdown of MFN2 in HEI-OC1 cells, the proportion of senescent cells increased significantly, and the protective effects of dbcAMP on cellular senescence and inflammatory phenotypes were markedly weakened. These findings suggested that cAMP may alleviate cochlear HC aging by enhancing MFN2-dependent mitochondrial fusion and homeostasis and by suppressing NF-κB signaling.

In 34- to 36-week-old C57BL/6J mice, after continuous intraperitoneal injection of dbcAMP for 28 days, loss of both IHCs and OHCs in the entire cochlea was reduced, and ABR threshold shifts at 8 kHz (10.63 ± 4.17 dB vs. 30.00 ± 5.48 dB) and 16 kHz (25.63 ± 6.78 dB vs. 36.67 ± 9.83 dB) were decreased, with a particularly marked reduction at 8 kHz. Besides, levels of the pro-inflammatory cytokines TNF-α and IL-6, as well as the expression of senescence-associated genes such as *p21* and *p16*, were reduced.

These findings demonstrated that treatment with dbcAMP could ameliorate ARHL, and its protective effects are closely associated with upregulation of MFN2 and suppression of the NF-κB inflammation pathway. However, since dbcAMP was administered systemically *in vivo*, indirect systemic effects cannot be completely excluded. Future studies are warranted to explore the employment of local administration methods, such as round window membrane application, or conditional genetic models. They also acknowledged that their mouse models carrying the *Cdh23* mutation and suggested further validation in other strains, such as CBA/CaJ, to strengthen the generalizability of conclusions. On the whole, dysregulation of mitochondrial dynamics is likely to play an important role in ARHL, mainly by disrupting mitochondrial morphology and activating inflammatory pathways in cochlear HCs. Both OPA1 and MFN2 exert protective effects by preserving mitochondrial morphology, although their mechanisms of action may differ. Accordingly, therapeutic strategies aimed at maintaining normal mitochondrial morphology, including SIRT3-mediated activation of OPA1 or enhancement of the cAMP/MFN2 pathway, may hold promise as targeted interventions for ARHL.

## 6 Conclusion

Recent studies have deepened our understanding of the molecular mechanisms underlying ARHL, particularly with respect to autophagy dysregulation, oxidative stress, stereocilia degeneration, and dysregulation of mitochondrial dynamics. These mechanisms facilitate the identification of potential therapeutic targets and intervention strategies for ARHL. Several molecules, including miR-34a, FOXG1, mTORC1, NRF2, HuR, OPA1, and MFN2, have emerged as promising therapeutic targets. However, the evidence supporting these targets varies in strength. Animal studies have provided support for miR-34a, FOXG1, mTORC1, NRF2, HuR, OPA1, and MFN2. In contrast, research on NMNAT1 has so far been mainly limited to HEI-OC1 cells and cochlear explants, with a lack of ARHL-specific *in vivo* validation.

Notably, these targets do not act in isolation but form an interconnected network that drives cochlear HC aging. mTORC1 overactivation could impair autophagy and exacerbate oxidative stress, thereby weakening protective pathways involving FOXG1 and NMNAT1. Oxidative stress and dysregulation of mitochondrial dynamics also mutually reinforce each other. Mitochondrial injury could further increase ROS, disrupt OPA1- and MFN2-regulated mitochondrial dynamics, and trigger inflammation, thereby accelerating loss of HCs. In addition, upstream regulators such as miR-34a and HuR may influence these processes through their roles in RNA stability and homeostasis.

Overall, research on ARHL therapeutic targets is shifting from a single-mechanism toward an integrated, multi-pathway perspective. Future studies should further define the interactions among these targets and develop multi-target therapeutic strategies that increase protective autophagy, reduce oxidative stress, preserve the structure of HC stereocilia, and maintain mitochondrial integrity, with the goal of delaying the progression of cochlear HC aging and ARHL.

Despite these advances, important barriers to clinical translation remain. First, most studies rely heavily on cell and mouse models, which may not fully represent changes of human cochlear. Future studies should therefore place greater emphasis on validation in large-animal models, such as non-human primates, pigs, or other species with cochlear structures and auditory ranges closer to humans. Second, the extensive use of C57BL/6 mice requires particular caution, because these mice carry the *Cdh23* gene, which causes early-onset hearing loss. Future studies should report the *Cdh23* genotype and further verify findings in C57BL/6 mice carrying the wild-type allele of *Cdh23*, such as B6.CAST-*Cdh23^Ahl^*, and other strains such as CBA/CaJ. Third, more standardized experimental designs are needed, including consistent animal strains, age windows, outcome measurements, and treatment approaches, to improve comparability across studies. Fourth, additional mechanistic work is required to clarify how these pathways interact *in vivo*, rather than examining each target in isolation. Finally, translational studies should focus more on inner-ear-specific drug delivery strategies, such as round window membrane administration and viral vectors with cochlear cell specificity, in order to enhance efficacy while minimizing systemic effects.

## References

[B1] Baird L. Yamamoto M. (2020). The molecular mechanisms regulating the KEAP1-NRF2 pathway. *Mol. Cell. Biol*. 40 e99–e20. 10.1128/MCB.00099-20 32284348 PMC7296212

[B2] Beer-Hammer S. Lee S. C. Mauriac S. A. Leiss V. Groh I. A. M. Novakovic A. et al. (2018). Gαi proteins are indispensable for hearing. *Cell. Physiol. Biochem*. 47 1509–1532. 10.1159/000490867 29940568 PMC11825972

[B3] Chadha S. Kamenov K. Cieza A. (2021). The world report on hearing, 2021. *Bull. World Health Organ.* 99 242A–242A. 10.2471/BLT.21.285643 33953438 PMC8085630

[B4] Ding R. Huang W. Shen C. Pan Y. Zhong Y. Kong B. et al. (2025). DLK/JNK3 upregulation aggravates hair cell senescence in mice cochleae via excessive autophagy. *Aging. Cell*. 24:e70099. 10.1111/acel.70099 40356329 PMC12341777

[B5] Ding Y. Meng W. Kong W. He Z. Chai R. (2020). The role of FoxG1 in the inner ear. *Front. Cell. Dev. Biol*. 8:614954. 10.3389/fcell.2020.614954 33344461 PMC7744801

[B6] Fitzian K. Brückner A. Brohée L. Zech R. Antoni C. Kiontke S. et al. (2021). TSC1 binding to lysosomal PIPs is required for TSC complex translocation and mTORC1 regulation. *Mol. Cell* 81 2705e–2721e. 10.1016/j.molcel.2021.04.019 33974911

[B7] Fu X. Sun X. Zhang L. Jin Y. Chai R. Yang L. et al. (2018). Tuberous sclerosis complex-mediated mTORC1 overactivation promotes age-related hearing loss. *J. Clin. Invest*. 128 4938–4955. 10.1172/JCI98058 30247156 PMC6205401

[B8] Glorieux C. Enríquez C. Buc Calderon P. (2025). The complex interplay between redox dysregulation and mTOR signaling pathway in cancer: A rationale for cancer treatment. *Biochem. Pharmacol*. 232:116729. 10.1016/j.bcp.2024.116729 39709038

[B9] Govindaraju S. Lee B. S. (2013). Adaptive and maladaptive expression of the mRNA regulatory protein HuR. *World J. Biol. Chem*. 4 111–118. 10.4331/wjbc.v4.i4.111 24340134 PMC3856306

[B10] Guo S. Cao J. Hong G. Song Y. Xia M. Li P. et al. (2025). mRNA metabolism regulator human antigen R (HuR) regulates age-related hearing loss in aged mice. *Nat. Aging*. 5 848–867. 10.1038/s43587-025-00860-y 40394214

[B11] He W. Wu F. Xiong H. Zeng J. Gao Y. Cai Z. et al. (2023). Promoting TFEB nuclear localization with curcumin analog C1 attenuates sensory hair cell injury and delays age-related hearing loss in C57BL/6 mice. *Neurotoxicology* 95 218–231. 10.1016/j.neuro.2023.02.004 36792013

[B12] He Z. H. Li M. Fang Q. J. Liao F. L. Zou S. Y. Wu X. et al. (2021). FOXG1 promotes aging inner ear hair cell survival through activation of the autophagy pathway. *Autophagy* 17 4341–4362. 10.1080/15548627.2021.1916194 34006186 PMC8726647

[B13] Henry W. S. Laszewski T. Tsang T. Beca F. Beck A. H. McAllister S. S. et al. (2017). Aspirin suppresses growth in PI3K-mutant breast cancer by activating AMPK and inhibiting mTORC1 signaling. *Cancer Res*. 77 790–801. 10.1158/0008-5472.CAN-16-2400 27940576 PMC5290090

[B14] Hu J. Xu M. Yuan J. Li B. Entenman S. Yu H. et al. (2016). Tauroursodeoxycholic acid prevents hearing loss and hair cell death in Cdh23(erl/erl) mice. *Neuroscience* 316 311–320. 10.1016/j.neuroscience.2015.12.050 26748055 PMC4724520

[B15] Jerome M. S. Kuthethur R. Kabekkodu S. P. Chakrabarty S. (2022). Regulation of mitochondrial function by forkhead transcription factors. *Biochimie* 198 96–108. 10.1016/j.biochi.2022.03.013 35367579

[B16] Jia Z. He Q. Shan C. Li F. (2018). Tauroursodeoxycholic acid attenuates gentamicin-induced cochlear hair cell death in vitro. *Toxicol. Lett*. 294 20–26. 10.1016/j.toxlet.2018.05.007 29751043

[B17] Jiang P. Ma X. Han S. Ma L. Ai J. Wu L. et al. (2022). Characterization of the microRNA transcriptomes and proteomics of cochlear tissue-derived small extracellular vesicles from mice of different ages after birth. *Cell. Mol. Life Sci*. 79:154. 10.1007/s00018-022-04164-x 35218422 PMC11072265

[B18] Johnson K. R. Tian C. Gagnon L. H. Jiang H. Ding D. Salvi R. (2017). Effects of Cdh23 single nucleotide substitutions on age-related hearing loss in C57BL/6 and 129S1/Sv mice and comparisons with congenic strains. *Sci. Rep*. 7:44450. 10.1038/srep44450 28287619 PMC5347380

[B19] Kim H. Choo O. S. Ha J. Yang J. Jang J. H. Park H. Y. et al. (2024). Objective and subjective efficacy of hearing aids in patients with mild-to-moderate unilateral hearing loss: A prospective study. *Eur. Arch. Otorhinolaryngol*. 281 1671–1681. 10.1007/s00405-023-08255-8 37803218

[B20] Klionsky D. J. Abdel-Aziz A. K. Abdelfatah S. Abdellatif M. Abdoli A. Abel S. et al. (2021). Guidelines for the use and interpretation of assays for monitoring autophagy (4th edition)1. *Autophagy* 17 1–382. 10.1080/15548627.2020.1797280 33634751 PMC7996087

[B21] Koffler-Brill T. Noy Y. Avraham K. B. (2023). The long and short: Non-coding RNAs in the mammalian inner ear. *Hear. Res*. 428:108666. 10.1016/j.heares.2022.108666 36566643 PMC9883734

[B22] Lee C. H. Park S. S. Lee D. H. Lee S. M. Kim M. Y. Choi B. Y. et al. (2020). Tauroursodeoxycholic acid attenuates cisplatin-induced hearing loss in rats. *Neurosci. Lett*. 722:134838. 10.1016/j.neulet.2020.134838 32061715

[B23] Li M. Zhao L. Liu J. Liu A. Jia C. Ma D. et al. (2010). Multi-mechanisms are involved in reactive oxygen species regulation of mTORC1 signaling. *Cell. Signal*. 22 1469–1476. 10.1016/j.cellsig.2010.05.015 20639120

[B24] Li P. Wang J. Zhao X. Ru J. Tian T. An Y. et al. (2020). PTEN inhibition attenuates endothelial cell apoptosis in coronary heart disease via modulating the AMPK-CREB-Mfn2-mitophagy signaling pathway. *J. Cell. Physiol*. 235 4878–4889. 10.1002/jcp.29366 31654396

[B25] Liu N. Landreh M. Cao K. Abe M. Hendriks G. J. Kennerdell J. R. et al. (2012). The microRNA miR-34 modulates ageing and neurodegeneration in Drosophila. *Nature* 482 519–523. 10.1038/nature10810 22343898 PMC3326599

[B26] Liu Y. Cao J. Cui J. Chen H. Qin S. Li Q. et al. (2025). cAMP-MFN2 signaling suppresses cochlear cell senescence and age-related hearing loss. *Front. Immunol*. 16:1715738. 10.3389/fimmu.2025.1715738 41383595 PMC12689285

[B27] Loughrey D. G. (2022). Is age-related hearing loss a potentially modifiable risk factor for dementia? *Lancet. Healthy Longev*. 3 e805–e806. 10.1016/S2666-7568(22)00252-5 36410369

[B28] Ma P. Wang S. Geng R. Gong Y. Li M. Xie D. et al. (2023). MiR-29a-deficiency causes thickening of the basilar membrane and age-related hearing loss by upregulating collagen IV and laminin. *Front. Cell. Neurosci*. 17:1191740. 10.3389/fncel.2023.1191740 37275774 PMC10232818

[B29] Mazurek B. Lou X. Olze H. Haupt H. Szczepek A. J. (2012). In vitro protection of auditory hair cells by salicylate from the gentamicin-induced but not neomycin-induced cell loss. *Neurosci. Lett*. 506 107–110. 10.1016/j.neulet.2011.10.060 22075224

[B30] Muñoz J. P. Basei F. L. Rojas M. L. Galvis D. Zorzano A. (2023). Mechanisms of modulation of mitochondrial architecture. *Biomolecules* 13:1225. 10.3390/biom13081225 37627290 PMC10452872

[B31] Nyenhuis S. B. Wu X. Strub M. P. Yim Y. I. Stanton A. E. Baena V. et al. (2023). OPA1 helical structures give perspective to mitochondrial dysfunction. *Nature* 620 1109–1116. 10.1038/s41586-023-06462-1 37612506 PMC12410014

[B32] Oh J. Youn C. K. Jun Y. Jo E. R. Cho S. I. (2020). Reduced mitophagy in the cochlea of aged C57BL/6J mice. *Exp. Gerontol*. 137:110946. 10.1016/j.exger.2020.110946 32387126

[B33] Oishi T. Matsumaru D. Ota N. Kitamura H. Zhang T. Honkura Y. et al. (2020). Activation of the NRF2 pathway in keap1-knockdown mice attenuates progression of age-related hearing loss. *NPJ Aging. Mech. Dis*. 6:14. 10.1038/s41514-020-00053-4 33318486 PMC7736866

[B34] Okur M. N. Sahbaz B. D. Kimura R. Manor U. Patel J. Park J. H. et al. (2023). Long-term NAD+ supplementation prevents the progression of age-related hearing loss in mice. *Aging. Cell*. 22:e13909. 10.1111/acel.13909 37395319 PMC10497810

[B35] O’Sullivan J. D. B. Bullen A. Mann Z. F. (2023). Mitochondrial form and function in hair cells. *Hear. Res*. 428:108660. 10.1016/j.heares.2022.108660 36525891 PMC10227193

[B36] Pang J. Xiong H. Lin P. Lai L. Yang H. Liu Y. et al. (2017). Activation of miR-34a impairs autophagic flux and promotes cochlear cell death via repressing ATG9A: Implications for age-related hearing loss. *Cell. Death. Dis*. 8:e3079. 10.1038/cddis.2017.462 28981097 PMC5680584

[B37] Rasmussen K. M. B. West N. C. Bille M. Sandvej M. G. Cayé-Thomasen P. (2022). Cochlear implantation improves both speech perception and patient-reported outcomes: A prospective follow-up study of treatment benefits among adult cochlear implant recipients. *J. Clin. Med*. 11:2257. 10.3390/jcm11082257 35456353 PMC9032498

[B38] Settembre C. Di Malta C. Polito V. A. Garcia Arencibia M. Vetrini F. Erdin S. et al. (2011). TFEB links autophagy to lysosomal biogenesis. *Science* 332 1429–1433. 10.1126/science.1204592 21617040 PMC3638014

[B39] Sha S. H. Qiu J. H. Schacht J. (2006). Aspirin to prevent gentamicin-induced hearing loss. *N Engl. J. Med*. 354 1856–1857. 10.1056/NEJMc053428 16641409

[B40] Tábara L. C. Segawa M. Prudent J. (2025). Molecular mechanisms of mitochondrial dynamics. *Nat. Rev. Mol. Cell. Biol*. 26 123–146. 10.1038/s41580-024-00785-1 39420231

[B41] Vélez-Ortega A. C. Freeman M. J. Indzhykulian A. A. Grossheim J. M. Frolenkov G. I. (2017). Mechanotransduction current is essential for stability of the transducing stereocilia in mammalian auditory hair cells. *Elife* 6:e24661. 10.7554/eLife.24661 28350294 PMC5407859

[B42] Wei Y. Yang W. Wu H. Kong M. Fan D. Zhang Y. et al. (2026). NMNAT1 activates autophagy to delay d-galactose-induced aging in cochlear hair cells. *Aging. Cell*. 25:e70373. 10.1111/acel.70373 41521645 PMC12793064

[B43] Xiong H. Pang J. Min X. Ye Y. Lai L. Zheng Y. (2022). miR-34a/ATG9A/TFEB signaling modulates autophagy in cochlear hair cells and correlates with age-related hearing loss. *Neuroscience* 491 98–109. 10.1016/j.neuroscience.2022.03.033 35367291

[B44] Xu S. Yang N. (2025). The role and research progress of mitochondria in sensorineural hearing loss. *Mol. Neurobiol*. 62 6913–6921. 10.1007/s12035-024-04470-4 39292339 PMC12078351

[B45] Yang J. Chen D. He Y. Meléndez A. Feng Z. Hong Q. et al. (2013). MiR-34 modulates caenorhabditis elegans lifespan via repressing the autophagy gene atg9. *Age* 35 11–22. 10.1007/s11357-011-9324-3 22081425 PMC3543738

[B46] Ying G. Zhao G. Xu X. Su S. Xie X. (2023). Association of age-related hearing loss with cognitive impairment and dementia: An umbrella review. *Front. Aging. Neurosci*. 15:1241224. 10.3389/fnagi.2023.1241224 37790283 PMC10543744

[B47] Yu R. C. Proctor D. Soni J. Pikett L. Livingston G. Lewis G. et al. (2024). Adult-onset hearing loss and incident cognitive impairment and dementia - A systematic review and meta-analysis of cohort studies. *Ageing. Res. Rev*. 98:102346. 10.1016/j.arr.2024.102346 38788800

[B48] Yun Q. Ma S. F. Zhang W. N. Gu M. Wang J. (2024). FoxG1 as a Potential therapeutic target for Alzheimer’s disease: Modulating NLRP3 inflammasome via AMPK/mTOR Autophagy Pathway. *Cell. Mol. Neurobiol*. 44:35. 10.1007/s10571-024-01467-4 38630150 PMC11023968

[B49] Zanfardino P. Amati A. Perrone M. Petruzzella V. (2025). The balance of MFN2 and OPA1 in mitochondrial dynamics, cellular homeostasis, and disease. *Biomolecules* 15:433. 10.3390/biom15030433 40149969 PMC11940761

[B50] Zhang A. Pan Y. Wang H. Ding R. Zou T. Guo D. et al. (2024). Excessive processing and acetylation of OPA1 aggravate age-related hearing loss via the dysregulation of mitochondrial dynamics. *Aging. Cell*. 23:e14091. 10.1111/acel.14091 38267829 PMC11019136

[B51] Zhang H. Xiao X. Pan Z. Dokudovskaya S. (2025). mTOR signaling networks: Mechanistic insights and translational frontiers in disease therapeutics. *Signal Transduct. Target Ther*. 10:428. 10.1038/s41392-025-02493-4 41469379 PMC12753737

[B52] Zhang J. Xiang H. Liu J. Chen Y. He R. R. Liu B. (2020). Mitochondrial sirtuin 3: New emerging biological function and therapeutic target. *Theranostics* 10 8315–8342. 10.7150/thno.45922 32724473 PMC7381741

[B53] Zhu Y. Lobato A. G. Zhai R. G. Pinto M. (2022). human nmnat1 promotes autophagic clearance of amyloid plaques in a drosophila model of Alzheimer’s disease. *Front. Aging. Neurosci*. 14:852972. 10.3389/fnagi.2022.852972 35401143 PMC8988035

[B54] Zou T. Xie R. Huang S. Lu D. Liu J. (2024). Potential role of modulating autophagy levels in sensorineural hearing loss. *Biochem. Pharmacol*. 222:116115. 10.1016/j.bcp.2024.116115 38460910

